# Infant dietary patterns and early childhood caries in a multi-ethnic Asian cohort

**DOI:** 10.1038/s41598-018-37183-5

**Published:** 2019-01-29

**Authors:** Shijia Hu, Yu Fan Sim, Jia Ying Toh, Seang Mei Saw, Keith M. Godfrey, Yap-Seng Chong, Fabian Yap, Yung Seng Lee, Lynette Pei-Chi Shek, Kok Hian Tan, Mary Foong-Fong Chong, Chin-Ying Stephen Hsu

**Affiliations:** 1 Faculty of Dentistry, National University of Singapore, Singapore, Singapore; 2 Singapore Institute for Clinical Sciences (SICS), Agency for Science, Technology and Research (A*STAR), Singapore, Singapore; 3 Saw Swee Hock School of Public Health, National University of Singapore, Singapore, Singapore; 4 Medical Research Council Lifecourse Epidemiology Unit and National Institute for Health Research Southampton Biomedical Research Centre, University of Southampton and University Hospital Southampton National Health Service Foundation Trust, Southampton, SO16 6YD UK; 5 Department of Obstetrics & Gynaecology, Yong Loo Lin School of Medicine, National University of Singapore, Singapore, Singapore; 6 Department of Paediatrics, KK Women’s and Children’s Hospital, Singapore, Singapore; 7 Department of Paediatrics, Duke-National University of Singapore Graduate Medical School, Singapore, Singapore; 8 Lee Kong Chian School of Medicine, Nanyang Technological University, Singapore, Singapore; 9 Departments of Paediatrics, Yong Loo Lin School of Medicine, National University of Singapore, Singapore, Singapore; 10 Division of Paediatric Endocrinology and Diabetes, Khoo Teck Puat-National University Children’s Medical Institute, National University Hospital, National University Health System, Singapore, Singapore; 11 Division of Paediatric Allergy, Immunology & Rheumatology, National University Hospital, Singapore, Singapore; 12 Department of Obstetrics & Gynaecology, KK Women’s and Children’s Hospital, Singapore, Singapore; 13 Clinical Nutrition Research Centre, Singapore Institute for Clinical Sciences, Agency for Science, Technology and Research, Singapore, Singapore

## Abstract

Dental caries, although preventable, remains one of the most prevalent chronic disease worldwide. Most studies focused on the relationship between sugar intake and caries. However, examining multidimensional dietary patterns is becoming increasingly important. Here, we examined the relationship between dietary patterns from ages 6 to 12 months and early childhood caries (ECC) at age 2 to 3-years. Infant dietary data was collected from caregivers and dietary pattern trajectories from 6 to 12 months derived. Oral examinations were carried out by trained calibrated dentists at ages 2 and 3 years. Associations between dietary pattern and ECC were estimated using generalized estimating equation. We found a 3.9 fold lower prevalence of decayed surfaces among children with high *Guidelines dietary pattern* scores at 6-months (IRR 0.26; CI [0.12–0.53]; p-value < 0.001) and 100% reduction of decayed surfaces with increased intakes of *Guidelines dietary pattern* foods from 6 to 12-month (IRR 2.4 × 10^−4^; CI [4.2 × 10^−7^–0.13]; p-value = 0.01). Suggesting that following the *Guideline dietary pattern*, which corresponds most closely to current World Health Organization weaning guidelines, at 6 months and an increase in pattern score between 6 and 12 months were protective against ECC development compared to *Predominantly breastmilk*, *Easy-to-prepare foods* and *Noodles (in soup) and seafood dietary patterns*.

## Introduction

Despite being a largely preventable disease, dental caries remains one of the most prevalent chronic disease worldwide^1^. Among American children 2 to 11 years of age, 42% experienced caries in their primary dentition, making it the most common chronic disease in children with signs that caries prevalence is increasing after decades of decline^2^. Dental caries is a dynamic process between protective and pathological factors with the major pathological pathway being the bacterial breakdown of fermentable dietary carbohydrates modified by the protective effects of salivary flow and fluoride intake^3^.

The notion that dietary sugar is a major factor in developing early childhood caries is well-known^4^. Despite the seemingly large number of studies examining the relationship between sugars and dental caries, only 4 out of the 55 studies included in the review looked specifically at the effect of dietary sugars in young children less than 3 years old^5–7^, with only 1 study examining children under age 1 year^8^. These studies show conflicting findings, with some showing a positive relationship^5,7,8^ and one showing no relationship between dietary sugars and dental caries in young children^6^. The impact of early prevention cannot be understated as it has been found that children with caries in their primary teeth are 3 times more likely to have caries in their permanent teeth as adults^9^.

Individuals do not consume nutrients or foods in isolation and investigating dietary patterns had the advantage of examining the overall diet which include the foods, food group, and nutrients; their combination and variety; and the frequency and quantity which they are habitually consumed^10^. It is possible to miss out on the effects of and interaction with other potentially contributory groups, when examining only single food group or nutrient for its impact on a disease.

Dietary patterns from 6 to 24 months of age are strongly influenced by mothers and other primary caregivers^11^. These patterns such as the *Guidelines dietary pattern*, and the *Western dietary pattern* have been associated with childhood and adolescent intelligent quotient^12,13^, and obesity^14^ respectively. The *Guidelines dietary pattern* corresponds most closely to current established weaning guidelines recommended by the World Health Organization^15^ and have been described in several studies across different populations^13,16–19^. The *Guidelines dietary pattern* is characterized by high consumption of rice porridge, fish and meat, fresh fruit and vegetables as core food items throughout the 6 to 12 months period^17^. Dietary patterns, a representation of the overall food intake pattern, may be associated with the risk of developing caries, although there are currently no known studies which have examined the relationship between dietary patterns and examining their impact on early childhood caries (ECC).

Singapore is a developed country which has 100% water fluoridation and a multi-ethnic Asian population. Although dental caries prevalence is comparable to other developed countries at 12 years old, ECC is disproportionally high at 40% among Singaporean preschool children^20^. This could in part be attributed to cultural differences in infant caregiving, which is often based on tradition rather than recommended guidelines. For example, a third of infants in a previous study had food added to their bottles and more than a third had sweetened beverages via a bottle, practices that increases frequency of exposure to fermentable carbohydrates^17^.

With that in mind, examining dietary pattern in young children may provide more information on feeding habits and the effect of dietary factors on the risk of early childhood caries than the examination of sugar intake alone^21^. This study is based on the Growing Up in Singapore Towards healthy Outcomes (GUSTO) cohort which is a prospective mother-offspring cohort study that collected longitudinal data on the offspring from birth^22^ until early childhood (6 years old). This study aims to investigate the relationship between infant dietary patterns at ages 6 months to 1 year and ECC in 2 to 3-year-old children, with the hypothesis that the different dietary patterns of the infants at 6 months to 1 year results in a difference in severity of ECC at 2 to 3 years.

## Materials and Methods

### The GUSTO cohort and study design

The GUSTO cohort is a mother-offspring cohort study that recruited over 1200 pregnant women aged 18–50 years delivering at the National University Hospital (NUH) and KK Women’s and Children’s Hospital (KKH) between June 2009 and September 2010. The participants were of self-identified homogenous ethnic background representing the 3 major ethnicities of Singapore: Chinese, Malay and Indian. Subjects were followed very closely from birth with detailed observations and samples collected to provide insights into the management and prevention of important diseases. The study received ethical approval from the institutional review boards of the National Healthcare Group and Singapore Health services. (DSRB reference D/09/021)(CIRB reference 2009/280/D) Informed consent was obtained from all participants and their legal guardians. Additionally, all experiments were performed in accordance with relevant guidelines and regulations.

This is a cohort study utilizing the GUSTO data where the various domains of the GUSTO study examined nested groups for different health parameters including nutrition and oral health. Demographics such as household income, maternal age, maternal education, and birthweight were recorded at the initial recruitment visit.

### Infant diet data – independent variable

Infant diet intake data was collected from mothers or caregivers by mailing 3-day food diaries (Supplemental Methods) prior to their 6-, 9-, and 12-months postnatal visits. In cases where diaries were returned incomplete, a 24-hr recall interview by trained personnel was collected during the visit using a 5-stage, multiple-pass interviewing technique^23^. Data from either the 24-hr recalls or 1-day record from food diaries were used for dietary analyses to increase the sample size. A correlation (r ranging from 0.123 to 0.820) of the 1-day record with the 2 other days using a subset of infants with complete 3-day food diaries has been previously established^21^.

Each food item from the records was then assigned to one of the 72 sub-food groups within the 18 food groups based on type of food or similarities on nutrient content, conceptually similar to previous studies^10^, was used to categorize the foods consumed locally. Among the food groups, the confectionary and sugar sweetened beverages (SSB) groups were selected for analyses in this study. The confectionary food group consist of food such as chocolates, sweets, ice-cream, puddings and jellies while SSB consists of fruit drinks, carbonated soft drinks, sweetened soya milk, traditional drinks and other sweetened drinks like honey mixed with water.

Dietary pattern trajectories were calculated using multi-level mixed models generating intercepts and gradients of various patterns as described by Smithers *et al*.^12^. Further details can be found in Lim *et al*.^21^. Briefly, dietary trajectories were empirically constructed by mapping dietary patterns extracted by exploratory factor analysis at 6-, 9- and 12-months. The dietary patterns across the three time-points were examined for their similarity in the types of foods and their loadings to ascertain their suitability to be modelled as trajectories. The mapping of each pattern to a trajectory was based on similar key constituent foods (with high loadings) found in the dietary patterns across the three time-points and this correspondingly determined the name of each trajectory. In this study, 4 dietary pattern trajectories observed in the participants namely: 1) *Predominantly breastmilk*, 2) *Guidelines*, 3) *Easy-to-prepare foods* and 4) *Noodles (in soup) and seafood*^21^.

The multi-level mixed model generates estimates of intercepts and gradients. The intercept reflects the trajectory score at the start point of each pattern (6-months of age), while the gradient denotes the rate of change in trajectory scores over time from 6-months to 12 months of age. For example, a high *Guidelines dietary pattern* intercept score indicates close adherence to the *Guidelines dietary pattern* at 6 months of age, while a high *Guidelines dietary pattern* gradient score indicate continued high adherence to the *Guidelines dietary pattern* from 6 to 12 months of age.

Infants following the *Predominantly breastmilk pattern*, had more breastmilk than formula milk with feeding of fresh fruits at 9-months, and consumption of bean-curd, ethnic breads and starchy vegetables at 12-months. The *Guidelines dietary pattern* had high consumption of rice porridge, fish and meat, fresh fruit and vegetables, and followed the currently recommended WHO weaning guidelines^15,16^. The infants who followed the *Easy-to-prepare pattern* had foods such as infant cereals, juices, cakes and biscuits. The *Noodles (in soup) and seafood pattern* was a uniquely Asian pattern that was characterized by Asian adult foods such as noodles accompanied by eggs, seafood, bean curd and dried preserved fruits^21^.

### Oral health data – primary outcome

Oral examinations were carried out by 3 trained calibrated dental professionals at ages 2 and 3 years (intraclass correlation coefficient, >0.80) using the modified International Caries Detection and Assessment System (ICDAS) criteria^24^. The participants were examined in the supine position using the knee-to-knee technique. Artificial illumination was used and tooth surfaces examined with sterile mouth mirror and blunt explorer. The tooth status was assessed by visual inspection, aided by tactile inspection if necessary after cleaning and drying of the surfaces with sterile gauze^25^. No radiographs were taken. Potential confounding factors in oral hygiene habits such as night-time bottle feeding, brushing frequency, and fluoride exposure were recorded as a self-administered questionnaire at the age 2 and age 3 oral examination.

As there is a lag time for the influence of dietary patterns to manifest into examinable changes in teeth, caries status at 2 to 3-years were examined for association with their dietary patterns profiled at 6 to 12-months.

### Data analysis

The primary outcome, ECC, was coded in 3 ways and analysed independently as a binary variable (present/absent), number of decayed teeth (dt) and number of decayed surfaces (ds) with white spot/cavitation lesions (International Caries Detection and Assessment System (ICDAS) II codes 2–6). An initial univariate analysis of food groups most likely to be associated with ECC, namely sugar confectionary and sugar sweetened beverages (SSB) was conducted using a logistic regression. The amount of sugar consumption was estimated from the diet diaries. Furthermore, marginal associations of dietary pattern trajectories with early childhood caries at ages 2 and 3 years were estimated using generalized estimating equation (GEE) with exchangeable working correlation matrix. Negative binomial family distribution with log link function was used. Incidence rate ratio (IRR) for each variable in the model was estimated with the use of Huber-White sandwich robust estimates for standard errors. Interactions between age and dietary trajectories were not included in the models as they were not statistical significant (p-value > 0.10) as shown by Wald test, indicating similar effects of dietary trajectories on ECC at 2 and 3 years. Confounding factors found in the literature such as socio-demographic characteristics, oral hygiene habits, perinatal and postnatal characteristics were adjusted in the analyses.

Significance level was set at p-value < 0.05. Statistical software STATA/SE Version 14 (StataCorp. 2015. Stata Statistical Software: Release 14. College Station, TX: StataCorp LP) was used to carried out all statistical analysis.

## Results

**Figure 1:**
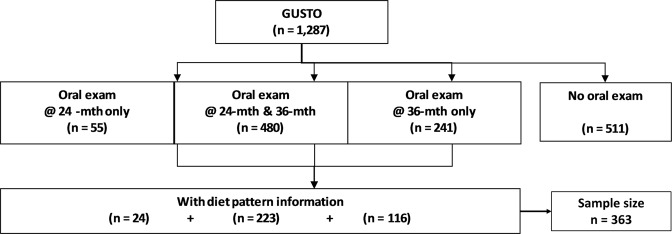
Summary of study subjects included from the GUSTO cohort.

In total, 776 children, out of the 1,287 children from GUSTO cohort had oral examinations at either 2 or 3 years old. Oral examinations were conducted on 535 children at age 2 years and 721 children at age 3 years. Unweighted prevalence of caries among children aged 24 and 36 months were noted at 17.8% and 42.9%, respectively. There were 363 children (46.8%) with dental examinations who also had infant feeding pattern data collected at the 6, 9, and 12 month postnatal visits (Fig. 1). These 363 subjects made up the final study sample involving ECC and dietary patterns.

Characteristics and ECC status of the subjects at 3 years old and sugary food sub-groups consumption were presented in Table 1. There were no difference in race, gender, maternal age, maternal education and household income between subjects with ECC and those without.

**Table 1:** Demographic Characteristics of subjects with Diet pattern information and Oral exam at 3 years old (n = 339) and Sugary food sub-groups consumption.

	No Caries at 3 Years Old	Caries at 3 Years old	P-value
	Frequency (%)	Frequency (%)	
Ethnicity			0.741
Indian	31 (15.8%)	19 (13.3%)	
Malay	52 (26.5%)	42 (29.4%)	
Chinese	113 (57.7%)	82 (57.3%)	
Gender			0.376
Male	96 (49.0%)	77 (53.8%)	
Female	100 (51.0%)	66 (46.2%)	
Mother’s age			0.574
<31 years old	94 (48.0%)	73 (51.0%)	
≥31 years old	102 (52.0%)	70 (49.0%)	
Mother’s education			0.610
No education/primary	6 (3.1%)	5 (3.5%)	
Secondary	68 (34.7%)	55 (38.5%)	
GCE	47 (24.0%)	38 (26.6%)	
University	74 (37.8%)	44 (30.8%)	
Household income			0.104
<$2,000	18 (9.2%)	25 (17.5%)	
$2,000–$3,999	61 (31.1%)	36 (25.2%)	
$4,000–$5,999	40 (20.4%)	36 (25.2%)	
>=$6,000	66 (33.7%)	40 (28.0%)	
Don’t know/Refused	10 (5.1%)	5 (3.5%)	
**Sugary food sub-groups consumption**			
	**Mean (SD)**	**Mean (SD)**	**P-value**
Confectionary (Grams per day)	2.22 (±3.51)	2.76 (±5.75)	0.345
Sugar Sweetened Beverages (Millilitres per day)	68.6 (±217)	49.5 (±105)	0.390

When analysed with ECC status at 3 years old, no significant association with frequency and amount of SSB with the presence or absence of ECC. The trend was similar in relation to confectionary consumption. (Table 2).

**Table 2:** Relationship between sugary food sub-groups consumption and early childhood caries.

Food group consumption per day	At 3 years old (n = 339)	
	ICDAS (caries present/absent)	
	OR (95% CI)	p-value
Confectionary		
Frequency	1.05 (0.25 to 4.43)	0.94
Amount in grams	1.03 (0.93 to 1.15)	0.54
Sugar Sweetened Beverages		
Frequency	0.67 (0.42 to 1.09)	0.11
Amount in millilitre	1.00 (0.99 to 1.00)	0.48

With regards to the hypothesis of the study, GEE analysis found that the Guidelines dietary pattern was significantly associated with the total number of tooth surfaces with white spot/cavitated lesions (ICDAS II codes 2–6). This was supported by the other 2 methods of coding the outcome variable ECC when examined independently. The *Guidelines dietary pattern* was significantly associated with each of the 3 methods of recording ECC (Table 3). This supports the hypothesis that different infant dietary patterns at 6 months to 1 year results in a difference in severity of ECC at 2 to 3 years.

**Table 3:** Association of dietary pattern trajectories with the 3 different methods of coding Early Childhood Caries before adjustment.

Subjects with Diet pattern information and Oral exam (n = 363)	ICDAS Caries present/absent		ICDAS decayed teeth (dt)		ICDAS decayed surface (ds)	
	OR (95% CI)	P-value	IRR (95% CI)	P-value	IRR (95% CI)	P-value
Age = 3 years old vs 2 years old	3.22 (2.32 to 4.47)	<0.001**	3.50 (2.50 to 4.90)	<0.001**	3.44 (2.45 to 4.83)	<0.001**
*Predominantly breastmilk* gradient	3.23 (0.03 to 360)	0.626	4.22 (0.05 to 380)	0.531	3.10 (0.07 to 146)	0.565
*Predominantly breastmilk* intercept	0.97 (0.74 to 1.28)	0.849	1.01 (0.79 to 1.28)	0.944	0.99 (0.79 to 1.25)	0.964
*Guidelines dietary pattern* gradient	0.51 (0.00 to 155)	0.817	0.08 (0.00 to 10.76)	0.313	0.08 (0.00 to 15.25)	0.341
*Guidelines dietary pattern* intercept	0.44 (0.21 to 0.92)	0.029**	0.37 (0.20 to 0.72)	0.003**	0.31 (0.16 to 0.06)	0.001**
*Easy-to-prepare* gradient	0.03 (0.00 to 50)	0.344	0.24 (0.00 to 206)	0.676	1.83 (0.00 to 6667)	0.885
Easy-to-prepare intercept	0.54 (0.17 to 1.74)	0.301	0.66 (0.21 to 2.03)	0.466	0.85 (0.21 to 3.51)	0.821
*Noodles and seafood* gradient	4042 (0.48 to 34200000)	0.072	506 (0.27 to 941189)	0.105	68 (0.01 to 336195)	0.331
*Noodles and seafood* intercept	1.19 (0.25 to 5.71)	0.832	1.73 (0.37 to 8.12)	0.490	1.22 (0.20 to 7.32)	0.824

After adjustment, significantly lower number of decayed tooth surfaces (ds) were found in Indian participants (p-value = 0.005) compared to Chinese and Malay participants. Controlling for ethnicity had little effects on the associations between a Guideline pattern and the dental outcomes. Additionally, ds was found to be higher in subjects who used fluoride toothpaste at 3 years (p-value = 0.014) when compared to subjects who did not. (Table 4).

**Table 4:** Association of dietary pattern trajectories with early childhood caries.

Subjects with Diet pattern information and Oral exam (n = 363)	ICDAS decayed surfaces (ds)		
	Adjusted Incidence Risk Ratio (IRR)	95% CI	P-value
Age = 3 years old Vs 2 years old	1.72	(1.00 to 2.96)	0.049**
*Predominantly breastmilk* gradient	9.80	(0.10 to 997.90)	0.333
*Predominantly breastmilk* intercept	1.20	(0.86 to 1.68)	0.276
*Guidelines dietary pattern* gradient	2.4 × 10^−4^	(4.2 × 10^−7^ to 0.13)	0.010**
*Guidelines dietary pattern* intercept	0.26	(0.12 to 0.53)	<0.001**
*Easy-to-prepare* gradient	0.28	(0.00 to 18836.43)	0.824
*Easy-to-prepare* intercept	0.69	(0.09 to 5.15)	0.715
*Noodles and seafood* gradient	4.36	(0.00 to 740989.20)	0.811
*Noodles and seafood* intercept	1.34	(0.20 to 9.07)	0.762
Ethinicity			0.005**
Indian	1.00	(reference)	
Malay	2.85	(1.29 to 6.28)	
Chinese	3.09	(1.40 to 6.79)	
Gender			0.530
Male	1.00	(reference)	
Female	0.86	(0.54 to 1.37)	
Mother’s Age			0.169
<31 years old	1.00	(reference)	
>=31 years old	1.50	(0.84 to 2.67)	
Mother’s Education			0.675
No Education/Primary	1.00	(reference)	
Secondary	1.37	(0.42 to 4.51)	
GCE	0.95	(0.28 to 3.14)	
University and above	0.93	(0.26 to 3.30)	
Household Income			0.101
<$2,000	1.00	(reference)	
$2,000–$3,999	0.47	(0.22 to 1.03)	
$4,000–$5,999	0.45	(0.20 to 1.00)	
>=$6,000	0.33	(0.14 to 0.77)	
Don’t know/refused	0.29	(0.09 to 0.89)	
Birth Weight			0.426
<2,500 g	1.00	(reference)	
>=2,500 g	1.66	(0.48 to 5.73)	
Night-time Bottle-feeding at 2-yr			0.334
No	1.00	(reference)	
Yes	1.32	(0.75 to 2.31)	
Brushing Frequency at 2-yr			0.882
None	1.00	(reference)	
Once	0.87	(0.38 to 1.97)	
>=Twice	0.83	(0.39 to 1.74)	
Fluoride Toothpaste used at 2-yr			0.797
No	1.00	(reference)	
Yes	0.78	(0.37 to 1.65)	
Don’t know	0.94	(0.35 to 2.52)	
Brushing Frequency at 3-yr			0.639
None	1.00	(reference)	
Once	1.36	(0.72 to 2.58)	
>=Twice	1.31	(0.67 to 2.55)	
Fluoride Toothpaste Used at 3-yr			0.014**
No	1.00	(reference)	
Yes	1.82	(1.22 to 2.72)	
Don’t know	1.60	(0.61 to 4.22)	

Compared to age 2 years, ds was estimated to be 1.72 times higher at age 3 years (p-value = 0.049). Children with a high *Guidelines dietary pattern* intercept score at age 6 months had significantly lower ds (p-value < 0.001) with an incidence risk ratio (IRR) of 0.26. This translates to an increase of ds by 3.9 folds per unit decrease in *Guidelines dietary pattern* intercept score. Increased intake of *Guidelines dietary pattern* food from 6 to 12 months (gradient) was also found to be associated with a lower ds (p-value = 0.010), with an almost 100% reduction in ds. There were no significant associations of the scores of *Predominantly breastmilk pattern* (p-value = 0.276), *Easy-to-prepare pattern* (p-value = 0.824), and *Noodles and seafood pattern* (p-value = 0.762) with the ds (Table 4 and Supplemental Fig. 1).

## Discussion

The GUSTO cohort has provided an opportunity to examine the relationship between multi-ethnic dietary patterns and the caries status of young Asian children. Key findings of this study include significantly lower decayed surfaces observed among children with high *Guidelines dietary pattern* score at age 6 month and among children who continue to adhere to *Guidelines dietary pattern* foods between ages 6 and 12 months which is further supported by significantly lower chance of developing caries and having lower number of decayed teeth. No association was observed with the other dietary patterns. This suggests a very strong protective effect of having a high *Guidelines dietary pattern* trajectory on developing ECC.

The study did not find very strong evidence for traditional paradigm of eating sugary foods causing tooth decay. A reason could be due to the early dietary data time points of less than age 12 months when children at that age not being likely to be given large amounts of dietary sugar. This could be seen from the low amount per day of sugary confectionary (<3 g a day). This is in line with one other study examining the diet of children below 1 year^6^. Additionally, the amount given can also be very variable from child to child as seen from the large standard deviation of SSB consumption. In this situation, examining the dietary pattern as a whole can provide a better indicator for risk of developing ECC at a later age instead of focusing on 1 or 2 food groups.

The *Guidelines dietary pattern* trajectory is characterized by increased exposure of the infant to a variety of food groups including fruits, vegetables, whole grains, poultry and fish^26–28^. Infants on a healthy “Guidelines” diet are also likely to be the ones consuming low sugary snacks in between meals such as fruits, vegetables, grains, dairy products. These options may replace sugary snack foods such as SSBs, confectionary which are known risk factors for ECC. Accordingly, Nunn *et al*. found that children aged 2 to 5 years with good eating practices as measured by the Healthy Eating Index (adherence to the Food Guide Pyramid) are less likely to have severe ECC^29^.

There appears to be a strong overlap between the foods that are good for general health and the foods that are good for dental health^30^, indicating that dentists and dietitians should collaborate and provide advice for healthy diets and healthy teeth. Dietary changes are more difficult once habits are established, suggesting the importance of encouraging caregivers to adhere better to established feeding guidelines early^21^. Moreover, maternal dietary pattern is a strong predictor of infant dietary patterns^11^. This suggests that the target of change should be focused on the primary caregiver and family much earlier than the current practice. By understanding the factors that influence dietary patterns and sugar consumption, more targeted and culturally-appropriate support and advice can be provided by health professionals and policy-makers to help improve dietary patterns as early as during pre-pregnancy counselling^31^. In addition to oral health promotion, this holistic dietary counselling can improve overall health and wellbeing by impacting obesity and other associated diseases^14^.

One of the factors affecting the effectiveness of caries prevention programs have been the early identification of children at high risk of ECC^32^. With 16% of the population accounting for 78% of the disease in Singapore^20^, this means that sometimes resources may not be directed at the areas where they can have the biggest impact. Using dietary pattern analysis as described presently, it can serve as a component in early identification of dietary risk contribution to better direct preventive efforts. This may improve reliability and validity over the current single food group analyses^4^.

As expected, the caries rate was higher at 3 years old when compared to at 2 years old as it is a chronic disease with a cumulative prevalence. The finding that Indian participants had lower rates of caries was similar to a previous study on a larger sample of the GUSTO cohort^25^. Interestingly, the use of fluoride toothpaste at 3 year old was associated with an increase in ECC. Similar to our previous report, this may be a result of an increased use of fluoride toothpaste due to recommendations from dentists after the child had caries in the first place^25^, a case of reverse causality.

Acknowledged limitation of our study is the self-reported dietary data that may introduce recall bias or social desirability bias^33^; however, the data was longitudinal in nature with good reproducibility in the records, mitigating the possible bias. Strengths of this study include the capture of important time points in the infant dietary pattern and their association with ECC in a large multi-ethnic Asian population cohort. Moreover, the longitudinal nature of the study allows for the influence of dietary patterns, profiled at 6 to 12-months, to manifest into examinable changes in teeth at 2 to 3-years. Additionally, important differences between the different cultures and ethnicities can be examined in a largely homogenous social structure.

## Conclusion

In conclusion, this is the first mother-offspring cohort study that investigated and found an association between dietary patterns and ECC. Following the *Guidelines dietary pattern* at 6 months and an increase in *Guidelines dietary pattern* score between 6 and 12 months were protective for the development of ECC compared to *Predominantly breastmilk, Easy-to-prepare foods* and *Noodles (in soup) and seafood* dietary patterns. Currently, there are no data on the early effects of dietary pattern on caries status in children. Our findings will aid development of a more holistic and earlier targeting dietary guidelines and policies for caries prevention alongside general health as the overall target. This may paradigm-shift our age-old adage of “don’t eat sweets” to “eat healthy food” to protect children’s teeth from cavities.

## Supplementary information

Supplementary Material

Supplemental Methods
