# Supplementary material for: Infant dietary patterns and early childhood caries in a multi-ethnic Asian cohort
Source: Sci Rep. 2019 Jan 29;9:852. doi: 10.1038/s41598-018-37183-5 (PMC6351619; doi:10.1038/s41598-018-37183-5)

Infant Dietary Patterns and Early Childhood Caries in a Multi-ethnic Asian Cohort

S Hu, YF Sim, JY Toh, SM Saw, KM Godfrey, YS Chong, F Yap, YS Lee, LPC Shek, KH Tan, MF Chong, CS Hsu

Supplementary Information

Appendix Figure legends

Supplemental Figure 1: Estimated DMFS at age 2 and 3 for each dietary pattern trajectories with adjustment for confounders

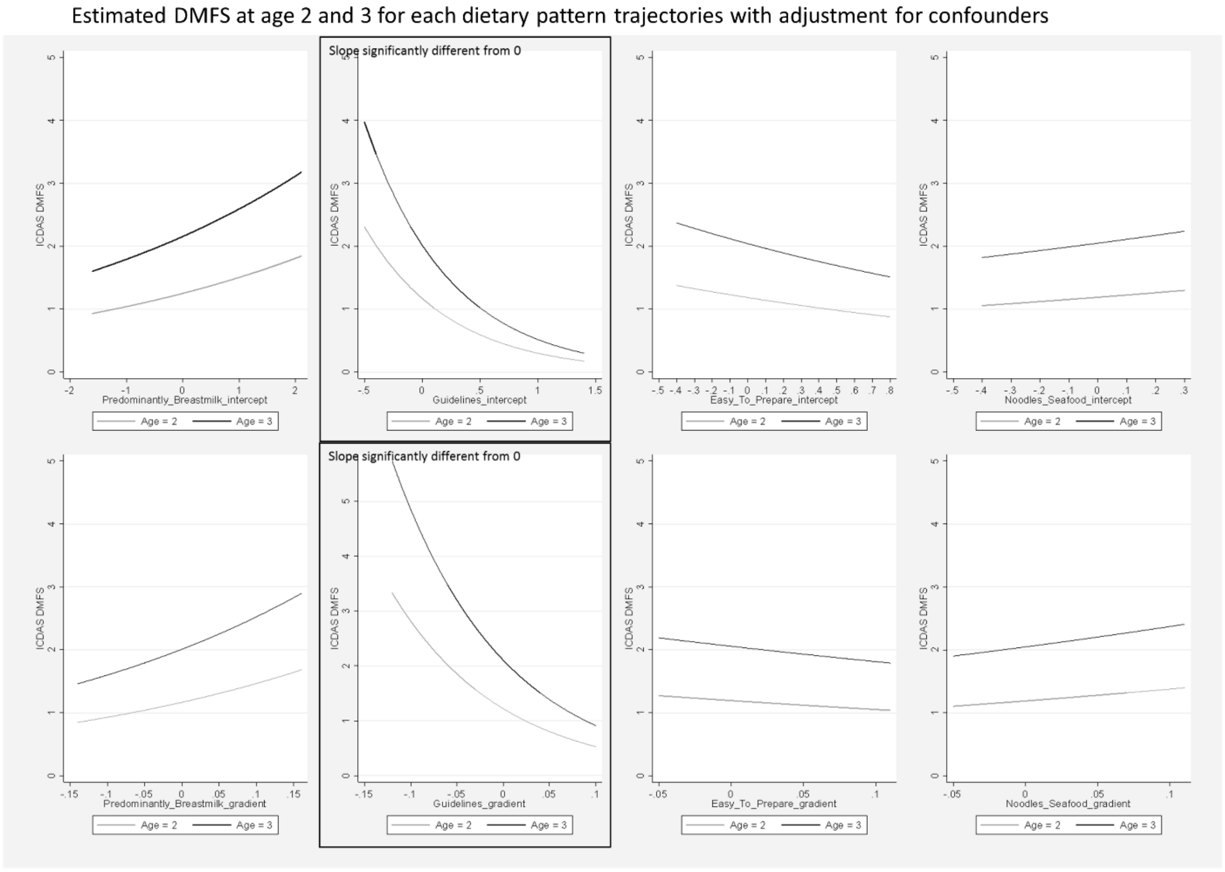

Supplement: Supplementary file 1 — Supplementary Material [file 41598_2018_37183_MOESM1_ESM.pdf]
