# Supplementary material for: Infant dietary patterns and early childhood caries in a multi-ethnic Asian cohort
Source: Sci Rep. 2019 Jan 29;9:852. doi: 10.1038/s41598-018-37183-5 (PMC6351619; doi:10.1038/s41598-018-37183-5)
Supplement: Supplementary file 2 — Supplemental Methods [file 41598_2018_37183_MOESM2_ESM.pdf]

Infant Dietary Patterns and Early Childhood Caries in a Multi-ethnic Asian Cohort

S Hu, YF Sim, JY Toh, SM Saw, KM Godfrey, YS Chong, F Yap, YS Lee, LPC Shek, KH Tan, MF Chong, CS Hsu

Supplemental Methods: Example of the 3-day food diary used in the infant diet data collection

Food items from the 24-h recalls or 1-day record (chosen by a randomized order) from the food diaries were grouped into pre-defined food groups. Food groups with low intakes among the infants (n < 3) were further combined based on similarity in culinary usage and nutrient profile. A total of 493, 894 and 1137 food items were identified and subsequently grouped into 34, 44 and 61 food groups at 6, 9 and 12 months of age, respectively. Dietary patterns at each time point were extracted by exploratory factor analysis (EFA) using the principal factor method in SPSS version 22.0 (IBM). EFA solutions were assessed for the magnitude of loadings of food groups. A dietary pattern score, standardized to a mean of zero and standard deviation of one, was calculated for each subject as a function of the contribution that each food made to the pattern.

3-DAY FOOD DIARY
FOR
INFANTS & CHILDREN

GUSTO

GROWING UP IN SINGAPORE TOWARDS HEALTHY OUTCOMES

GUSTO

GROWING UP IN SINGAPORE TOWARDS HEALTHY OUTCOMES

Content, design, layout & photography by:

GUSTO Nutrition Team

- **Dr *Mary Chong,* Investigator & Dietitian, GUSTO**
- **Dr *Han Wee Meng,* Investigator & Dietitian, GUSTO**
- **Dr *Low Yen Ling,* Investigator & Dietitian, GUSTO**
- **Ms *Doris Loh,* Investigator & Lactation Consultant (IBCLC), GUSTO**
- **Ms *Moira Khaw,* Communications Manager, GUSTO**
- **Ms *Marjorelee T. Colega,* Nutrition Research Assistant, GUSTO**

1st edition September 2010

© 2010 National University of Singapore
© 2010 Singapore Institute of Clinical Sciences
All rights reserved

Instructions:

Please write down every food your child takes for 3 days using this food diary. The 3 days should include 2 weekdays and 1 weekend (either Saturday or Sunday).

Completing this diary carefully will take you some time. But the records given by you will provide very valuable information to study the effects of diet on health.

It is important that you do not change the food and drinks that you give to your child when keeping this record. Please continue to give food items in the way that your child is usually fed.

Record all the foods that your child has eaten and drunk including snacks. Record everything at the time of eating, not from memory at the end of the day. Please write down the approximate time of eating and indicate whether the food or drink was prepared at home or purchased outside.

On the next two pages is a list of common foods and drinks. Next to each item are the sort of information we need to know. Please give as much detail as you can. An example is shown on page 4 .

On pages 5 to 8, you will find a series of food pictures. For some foods, you may find it easier to describe how much you gave to your child by comparing it to one of the pictures. You can also use the pictures of the cups, bowls and spoons to help indicate the amount you gave. For packaged foods, please write down the weights printed on the packaging to show the amount you provided for your child.

If you have any queries, please call ___________________________________
during 8:30 am to 6 pm, Monday to Friday.

Please return the diary to us after completion.
Thank you very much for your cooperation in completing such detailed diary.

Food/Drink	Description & Preparation	Amount
Biscuits	Plain, sweet, savoury, cream, chocolate, cheese, wafer, home-made. Give brand if possible.	Number.
Bread	White, wholemeal or fruit; thick, medium or thin slices. Give brand if possible. Write down type of fillings for bread buns.	Number of slices or buns.
Baby's instant cereal	Rice, wheat or oat cereal; plain or mix with other ingredients e.g. banana, vegetables, milk, chicken. Give brand name if possible.	Number of spoons. Refer to picture for size of spoon.
Baby's puree	Specify type e.g. vegetable, fruit; homemade or instant. Give brand name if possible.	Number of spoons. Refer to picture for size of spoon.
Breakfast cereal	With or without milk. Give brand name if possible.	Number of spoons. Refer to picture for size of spoon.
Cake/Kueh	Specify type; with or without coconut.	Number of slices. Refer to pictures (o1-o2) for size of slice.
Chicken	Drumstick, breast, wing or other parts; with or without skin or sauce; method of cooking and how it was presented e.g. pureed, minced.	Amount or refer to picture.
Cooking oil or salad dressing	Give type and brand if possible.	Number of spoons. Refer to picture for size of spoon.
Drinks	Specify type and brand e.g. Milo, Ovaltine, barley water; with or without ice.	State volume in mls.
Egg	Egg yolk, egg white or whole egg; method of cooking.	Number.
Fish	Specify type; method of cooking and how it was presented.	Number of spoons or slices. Refer to pictures (f1 to f3).
Fruit	Specify type and variety e.g. Fuji apple; with or without skin; method of preparation e.g. mashed, scraped, pureed.	Amount or number. Indicate whether small, medium or large size.
Fruit juice	Sweetened or unsweetened, fresh juice or cordial, diluted or undiluted. Give brand if possible.	State volume in mls.
Ice cream	Specify flavour and variety. Give brand name if possible.	Scoops or spoons. Refer to picture for size of spoon.
Margarine, jam, or other spreads	Specify type and brand if possible.	Number of spoons. Refer to picture for size of spoon.
Meats	Specify type; lean or fatty; method of cooking and how it was presented.	Amount or refer to pictures (g1 to g3).

Food/Drink	Description & Preparation	Amount
Milk	Formula milk (specify scoops given), breast milk, cow's milk, soya milk. Give brand name if possible.	State volume in mls.
Milk products	Examples include yogurt, cheese, cultured milk products. Specify type and brand if possible, e.g. Yakult, Vitagen; plain, flavored or unflavored; regular, low fat, soft.	Packed weight.
Noodles	Specify type e.g. bee hoon, mee sua, mifen, kway teow; method of cooking – soup, dry or fried.	Refer to pictures (e1 to e3).
Nuts	Specify type and brand if possible e.g., peanut, almond; dry roasted, deep fry; salted or unsalted.	Packet weight.
Porridge	Plain or cooked with ingredients. Thick or thin consistency.	Refer to pictures (c1 to c3).
Potato & Root Tubers	Examples include sweet potato, potato, yam; method of cooking and how it was presented e.g. boiled, deep fried, mashed, pureed.	Amount. Refer to pictures.
Pulses	Examples include beans, lentils/dhal, peas. Specify type and method of cooking.	Number of spoons.
Rice	White or brown or mixed; boiled or fried.	Refer to pictures (d1-d3).
Sausage	Specify type and size e.g. pork, chicken, beef; big or small.	Number.
Seeds	Examples include sesame seed, pumpkin seed.	Number of spoons.
Soup	Specify type; canned or home-made - list ingredients eaten.	Refer to pictures of bowls.
Snack and tidbits	Give brand and type.	Packet weight.
Sugar	White or brown sugar or sweetener.	Heaped or level spoons.
Sweets	Specify type e.g. toffee or boiled sweets. Give brand name if possible.	Number.
Tofu or tau kwa	Specify type; method of cooking; with or without soup.	Amount. Refer to pictures (f1 to f3).
Vegetables	Specify type and variety; method of cooking e.g. boiled, stewed and how it was presented e.g. chopped, minced, shredded or pureed.	Refer to pictures.
Meals eaten outside	Specify type e.g. Chinese, Indian, Japanese, fast food. Write down the name of the dish, cooking method (e.g. stir fried, grilled or steamed) and give ingredients where possible. Give the name of restaurant if it is a well-known chain.	Refer to pictures for portion sizes.

Example

Time	Placed Prepared<br>*H=Home*<br>*O=Outside*	Food /<br>Drink	Description and Preparation	Amount
8 am	*H*	*Milk*	*Brand XX Growing Up Milk*	*4 scoops in 240ml water*
10 am	*H*	*Fruit*<br>*Water*	*Ripe banana, mashed*<br>*Plain water*	*m2*<br>*150ml*
12 pm	*H*	*Infant Cereal*<br>*Meat*<br>*Vegetable*<br>*Oil*<br>*Water*	*4 dessertspoons of Brand XX Baby Cereal mixed with 120ml of water*<br>*Minced steamed chicken (from drumstick)*<br>*Pureed steamed carrots*<br>*Olive Oil*<br>*Plain water*	*1 R3 bowl*<br>*1 tablespoon*<br>*2 tablespoons*<br>*1 teaspoon*<br>*150ml*
2 pm	*H*	*Milk*	*Brand XX Growing Up Milk*	*4 scoops in 240ml water*
4 pm	*H*	*Rusk*<br>*Juice*	*Brand XX Baby Rusk*<br>*Brand XX Baby Juice (Apple Flavor)*	*2 pc.*<br>*150ml*
7 pm	*O*	*Fish Porridge*<br>*Vegetable*<br>*Water*	*Rice porridge with*<br>*Sliced threadfin fish and*<br>*Spinach (no oil added)*<br>*Plain water*	*c2*<br>*f1*<br>*1 tablespoon*<br>*150ml*
9:30 pm	*H*	*Milk*	*Brand XX Growing Up Milk*	*240ml*

Use the pictures below to help you indicate the amount of food your child has eaten. The pictures can also be used for foods not shown. Record the picture number or the dessertspoon (dsp) equivalence (=) of the portion size eaten.

The plate shown is a 9-inch plate with an inner 6-inch circle (typical size of a child's plate). The bowl shown is Rc (4 ½ -inch in diameter; typical size of a child's bowl).

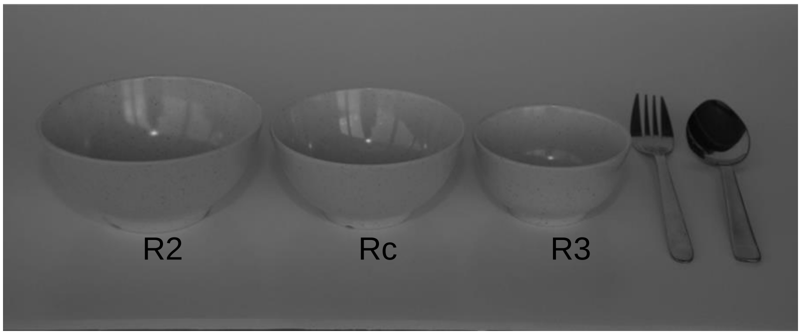

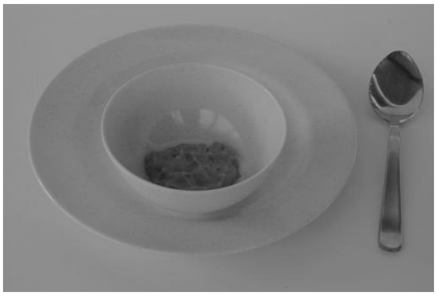
a1 = 1 dsp

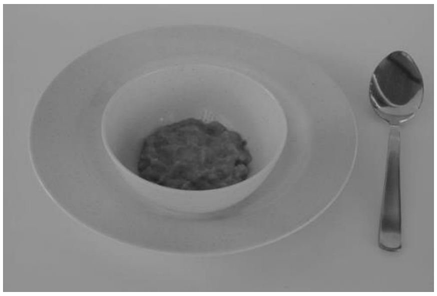
a2 = 2 dsp

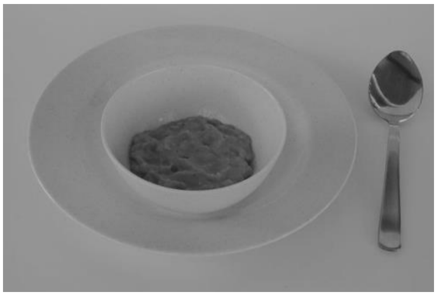
a3 = 3 dsp

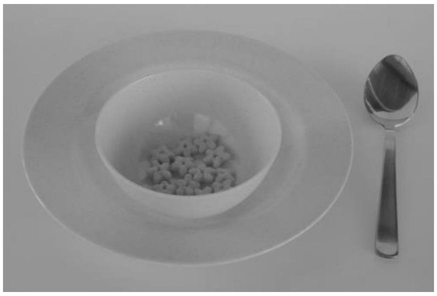
b1 = 1 dsp

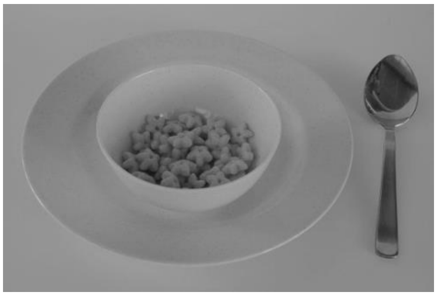
b2 = 3 dsp

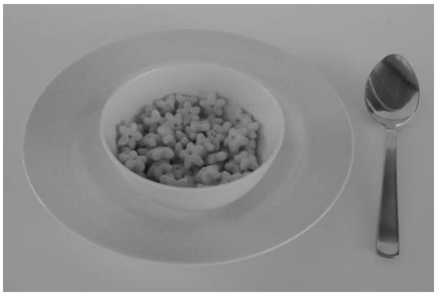
b3 = 5 dsp

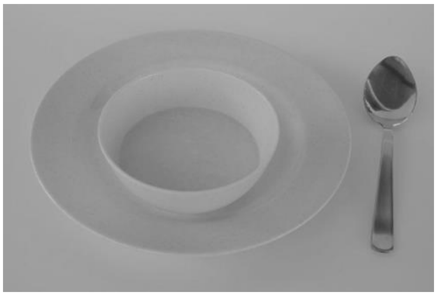
c1 = 4 dsp

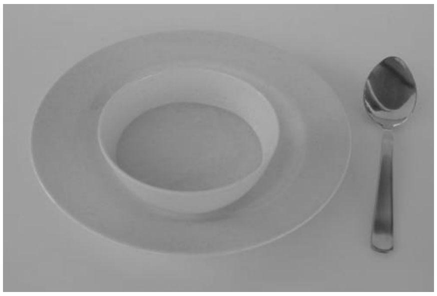
c2 = 6 dsp

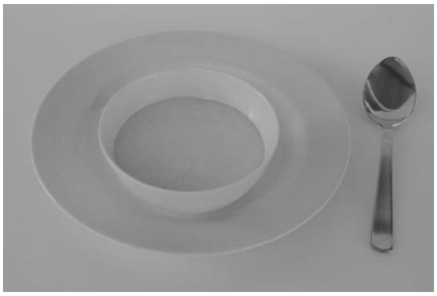
c3 = 10 dsp

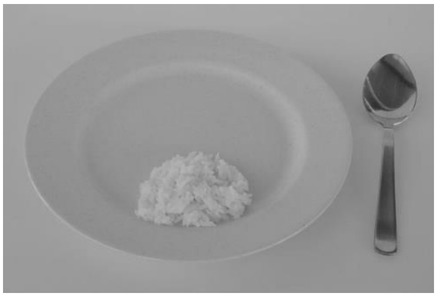
d1 = 2 dsp

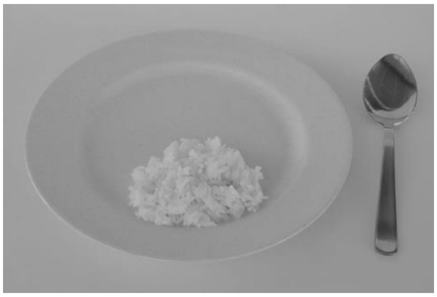
d2 = 3 dsp

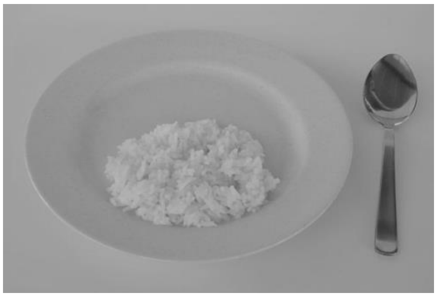
d3 = 5 dsp

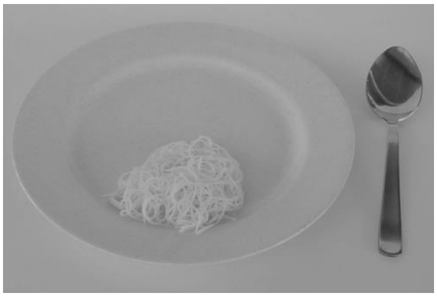
e1 = 2 dsp

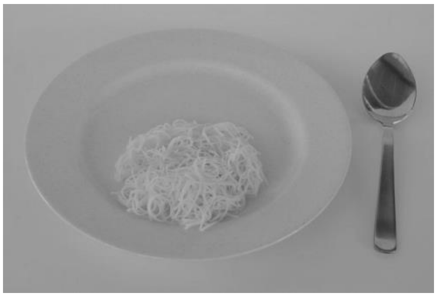
e2 = 3 dsp

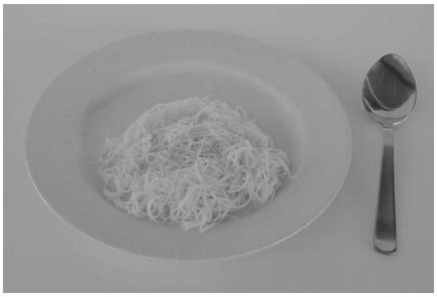
e3 = 5 dsp

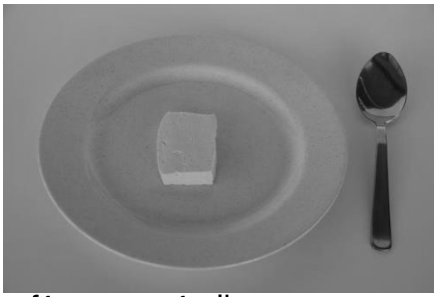
f1 = 1 slice

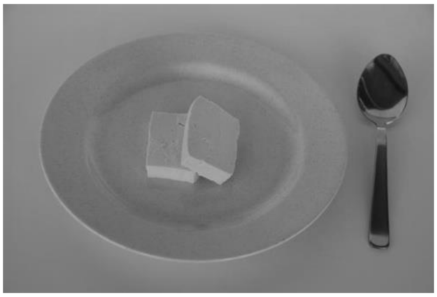
f2 = 2 slice

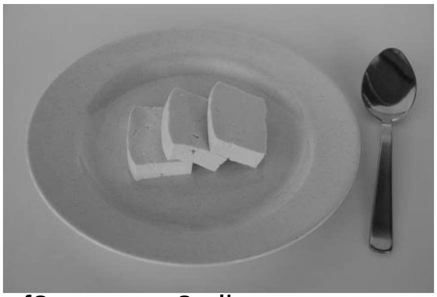
f3 = 3 slices

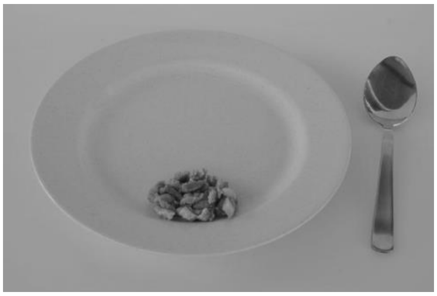
g1 = 1 dsp

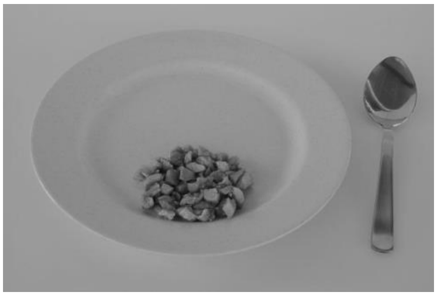
g2 = 2 dsp

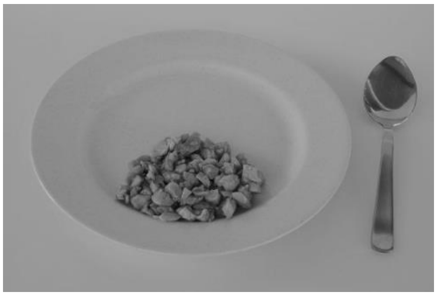
g3 = 3 dsp

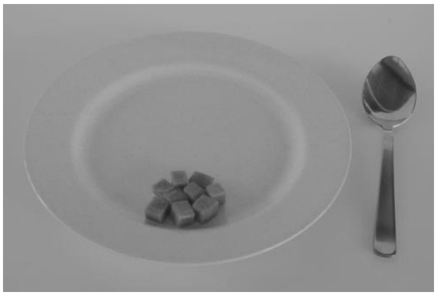
h1 = 1 dsp

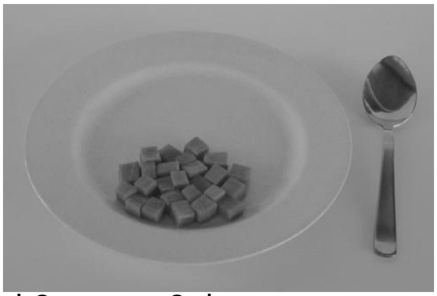
h2 = 3 dsp

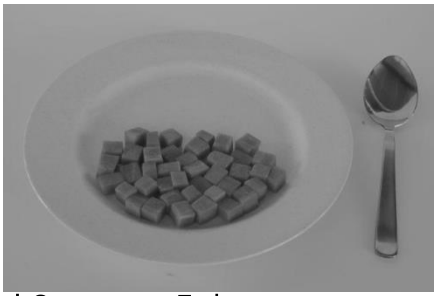
h3 = 5 dsp

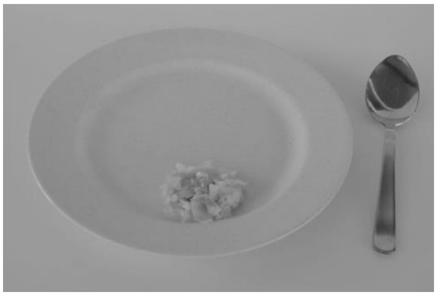

j1 = 1 dsp

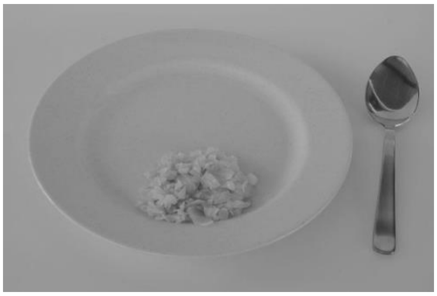

j2 = 2 dsp

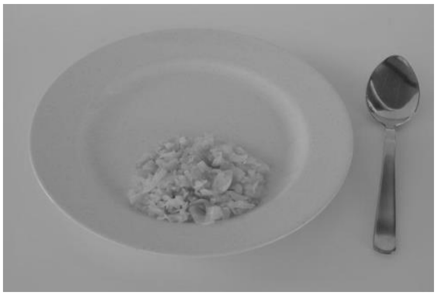

j3 = 3 dsp

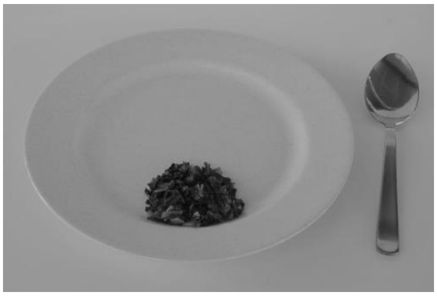

k1 = 1 dsp

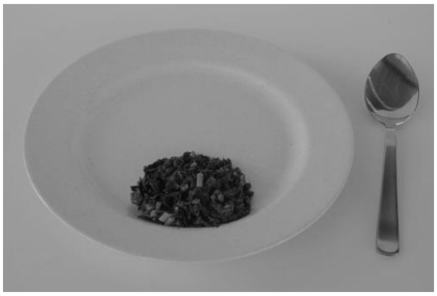

k2 = 2 dsp

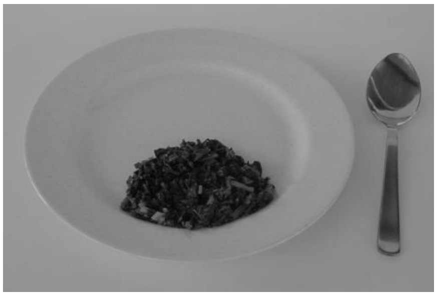

k3 = 3dsp

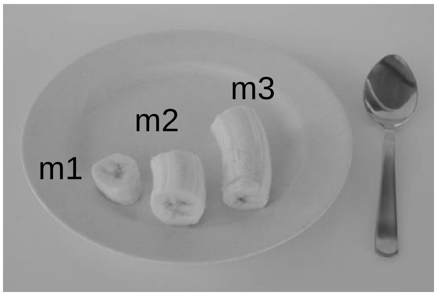

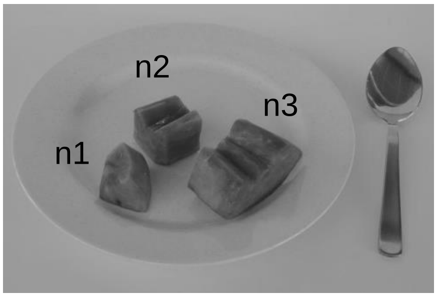

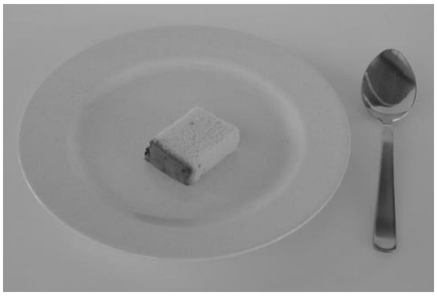

o1 = ½ slice

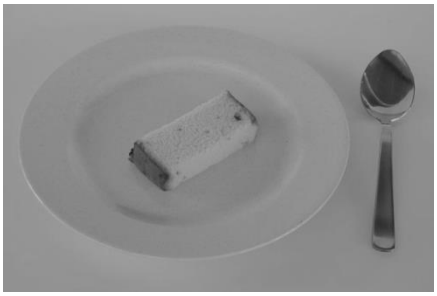

o2 = 1 slice

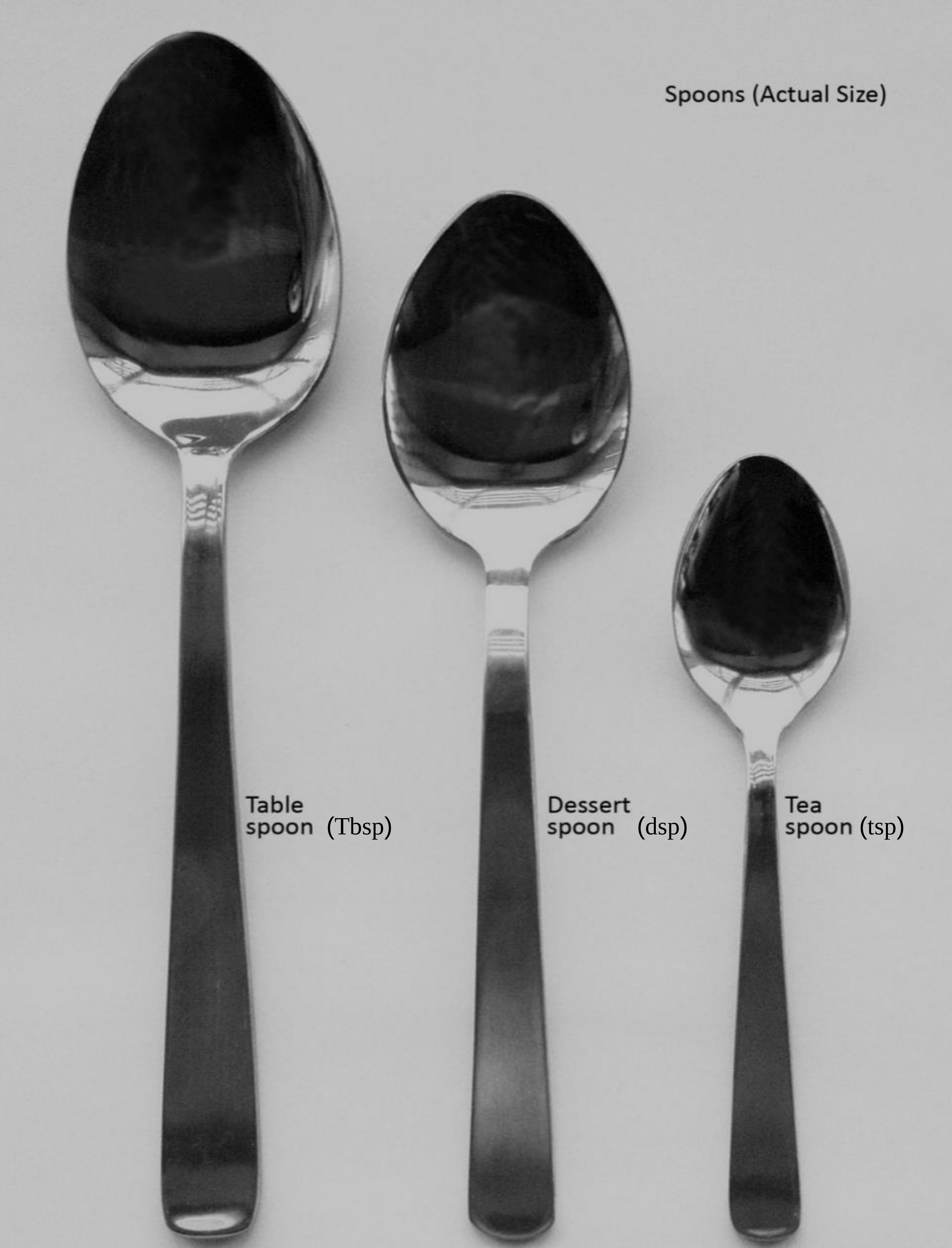
Spoons (Actual Size)
Table spoon (Tbsp)
Dessert spoon (dsp)
Tea spoon (tsp)

Date:_ _ / _ _ / 20_ _ Day of week:________________

DD MM YY

Time	Place prepared<br>H=Home<br>O=Outside	Food/Drink	Description & Preparation	Amount

Please check:

-Did you leave out anything e.g. drinks, fruits, sweets, chocolates, biscuits, other snacks, ice-cream, etc?

-Did you remember to write down any supplement your child took?

Date:_ _ / _ _ / 20_ _ Day of week:________________

DD MM YY

Time	Place prepared<br>H=Home<br>O=Outside	Food/Drink	Description & Preparation	Amount

Please check:

-Did you leave out anything e.g. drinks, fruits, sweets, chocolates, biscuits, other snacks, ice-cream, etc?

-Did you remember to write down any supplement your child took?

Date:_ _ / _ _ / 20_ _ Day of week:________________

DD MM YY

Time	Place prepared<br>H=Home<br>O=Outside	Food/Drink	Description & Preparation	Amount

Please check:

-Did you leave out anything e.g. drinks, fruits, sweets, chocolates, biscuits, other snacks, ice-cream, etc?

-Did you remember to write down any supplement your child took?

Date:_ _ / _ _ / 20_ _
DD MM YY

Day of week:________________

Time	Place prepared<br>H=Home<br>O=Outside	Food/Drink	Description & Preparation	Amount

Please check:

-Did you leave out anything e.g. drinks, fruits, sweets, chocolates, biscuits, other snacks, ice-cream, etc?

-Did you remember to write down any supplement your child took?

Date:_ _ / _ _ / 20_ _ Day of week:________________

DD MM YY

**Time**	**Place prepared**<br>**H=Home**<br>**O=Outside**	**Food/Drink**	**Description & Preparation**	**Amount**

Please check:

-Did you leave out anything e.g. drinks, fruits, sweets, chocolates, biscuits, other snacks, ice-cream, etc?

-Did you remember to write down any supplement your child took?

Date:_ _ / _ _ / 20_ _   Day of week:________________

DD MM YY

**Time**	**Place prepared**<br>**H=Home**<br>**O=Outside**	**Food/Drink**	**Description & Preparation**	**Amount**

Please check:

-Did you leave out anything e.g. drinks, fruits, sweets, chocolates, biscuits, other snacks, ice-cream, etc?

-Did you remember to write down any supplement your child took?

General Questions: Please circle your answers.

1. What type of oil/fat do you use for cooking for your child (stir-frying, pan frying,deep frying, stewing)?

a. Blended vegetable oil/ palm oil
b. Corn oil
c. Soya bean oil
d. Sunflower oil
e. Safflower oil
f. Wheat germ oil
g. Olive oil
h. Peanut oil
i. Canola oil
j. Lard, ghee, tallow, dripping or any other animal fat
k. Cooking margarine, butter shortening
l. Others, please specify
m. Don't know
n. Do not cook at home at all

2. What type of bread do you usually give to your child?

a. White bread (please state brand and type______________________________)
b. Wholemeal bread (please state brand and type __________________________)
c. A mixture of answers 1 and 2
d. Others (please state brand and type ___________________________________)
e. Do not give bread at all

3. What kind of spread do you usually use on bread or crackers for your child?

a. Butter (please state brand and type ____________________________________)
b. Hard margarine (please state brand and type _____________________________)
c. Soft margarine (please state brand and type ______________________________)
d. Kaya
e. Peanut butter
f. Cheese spread (please state brand and type ______________________________)
g. Others (please state brand and type ____________________________________)
h. Do not use spread at all (Go to question 5)

4. How thickly do you spread margarine, butter or other spreads on the bread or crackers for your child?

a. Thickly spread
b. Medium
c. Thinly spread

5. Aside from breast milk / formula milk, what type of milk do you usually give to your child?

a. Whole/full cream milk
b. Low fat
c. Skimmed/non-fat
d. Sweetened condensed milk
e. Others (please specify____)
f. Do not give milk at all

6. When your child eats meat (e.g. beef, mutton, pork etc.), does he usually eat:

a. All the fat
b. Some of the fat
c. None of the fat
d. Do not give meat at all

7. When your child eats poultry (e.g. chicken, duck), does he usually eat:

a. All the skin
b. Some of the skin
c. None of the skin
d. Do not give poultry at all

8. At the table, do you usually add salt, soya sauce or gravy to your child's food?

a. Yes
b. No

9. When your child eats fruits such as apple, pear, plum, grape, etc., does he eat the skin?

a. Yes
b. No
c. Do not eat these fruits.

Notes

For more information, please contact:

The GUSTO Team

Developmental Origins: Singapore (DevOS)
Department of Obstetrics and Gynaecology
National University Singapore
National University Health System
NUHS Tower Block, Level 12
1E Kent Ridge Road
Singapore 119228

Tel: 93678944
Email: info@gusto.sg
www.gusto.sg

Collaborators:

KK Women's and Children's Hospital
SingHealth

NUH
National University Hospital

A*STAR
Singapore Institute for Clinical Sciences

Administered by:

NUHS
National University Health System

NUS
National University of Singapore
Yong Loo Lin School of Medicine

This study is supported by MUIS.
